# Evaluation of portal pressure by doppler ultrasound in patients with cirrhosis before and after simvastatin administration – a randomized controlled trial

**DOI:** 10.12688/f1000research.13915.1

**Published:** 2018-03-01

**Authors:** Nadia Elwan, Raafat Salah, Manal Hamisa, Ebtsam Shady, Nehad Hawash, Sherief Abd-Elsalam

**Affiliations:** 1 Department of Tropical Medicine, Faculty of Medicine, Tanta University, Tanta, Gharbia Governorate, Egypt; 2 Department of Radiology, Tanta University, Tanta, Gharbia Governorate, Egypt

**Keywords:** Simvastatin; Portal hypertension; Cirrhosis; Doppler; Ultrasound.

## Abstract

**Background:** Portal hypertension is one of the most frequent complications of cirrhosis. β-adrenergic blockers, with or without organic nitrates, are currently used as hypotensive agents. Statins such as simvastatin seem to be safe for patients with chronic liver diseases and exert multiple pleiotropic actions. This study aimed to assess PTH using Doppler ultrasound in patients with cirrhosis before and after simvastatin administration.

**Methods:** This randomized controlled clinical trial was conducted on 40 patients with cirrhosis who were randomized into 2 groups: group I included 20 patients with cirrhosis who were administered 20 mg of simvastatin daily for 2 weeks and then 40 mg daily for another 2 weeks, and group II included 20 patients with cirrhosis who did not receive simvastatin as a control group. All patients underwent full clinical examination, laboratory investigations, and abdominal Doppler ultrasound at baseline and after 30 days to evaluate portal vein diameter, blood flow volume, direction and velocity of portal vein blood flow, hepatic artery resistance and pulsatility indices, splenic artery resistance index, portal hypertension index (PHI), liver vascular index, and modified liver vascular index (MLVI).

**Results: **There was a highly significant decrease in the hepatic artery resistance index  in group I, from 0.785 ± 0.088 to 0.717 ± 0.086 (P < 0.001). There was a significant decrease in the PHI in group I , from 3.915 ± 0.973 m/sec to 3.605 ± 1.168 m/sec (P = 0.024). Additionally, there was a significant increase in the MLVI in group I from 11.540 ± 3.266 cm/sec to 13.305 ± 3.222 cm/sec, an increase of 15.3% from baseline (P = 0.009). No significant adverse effects were detected.

**Conclusions:** Simvastatin is safe and effective in lowering portal hypertension.

[ClinicalTrials.gov Identifier: NCT02994485]

## Introduction

Liver cirrhosis is considered to be the most common cause of portal hypertension (PHT) ^1^. Increased portal inflow and increased outflow resistance are associated with the development of PHT ^2^. Liver transplantation is indicated in patients with advanced cirrhosis complicated by PHT; furthermore, morbidity and mortality are increased in these patients ^3, 4^.

The ideal hypotensive drug for PHT should decrease portal pressure by lowering intrahepatic vascular resistance while maintaining or increasing hepatic blood flow ^3^. Moreover, it should improve liver function through its antifibrotic effects, and it should be able to increase nitric oxide bioavailability in the liver to help fulfill many of these requirements ^3, 5– 8^.

Currently, the available therapies for PHT are based on the use of β-adrenergic blockers, with or without organic nitrates, and allow achievement of the target hemodynamic response in less than half of patients. Moreover, about 30% of patients may have contraindications or may not tolerate β-blockers ^9^.

Statins such as simvastatin are used mainly for cardiovascular diseases and metabolic syndrome. They exert multiple pleiotropic effects and can be used safely in patients with chronic liver diseases ^10^. They decrease Rho-kinase activity in activated hepatic stellate cells ^11^. In addition, statins have anti-inflammatory, immunomodulatory, and antioxidant properties ^12^. Simvastatin is also known to induce Krüppel-like factor 2, which improves liver fibrosis and PHT by increasing nitric oxide bioavailability ^13^.

Color Doppler ultrasound is an important non-invasive tool that can be used to record portal venous system blood flow ^14^. This study aimed to evaluate PHT by Doppler ultrasound in patients with cirrhosis before and after simvastatin administration.

## Patients and Methods

This randomized controlled study was conducted in the Department of Tropical Medicine at Tanta University Hospital. Forty patients with cirrhosis and PHT were enrolled from April to November 2016. All patients provided written informed consent, and the study was approved by the Ethics Committee of the Faculty of Medicine at Tanta University. All patients had code numbers to ensure anonymity. The study was registered on www.clinicaltrials.gov (ClinicalTrials.gov Identifier: NCT02994485).

Patients diagnosed with liver cirrhosis by ultrasound (a coarse echogenic pattern, surface irregularity, attenuated hepatic veins, and a bulky caudate lobe) who also had clinical signs of PHT (esophageal varices, splenomegaly, ascites, and grade I-II hepatic encephalopathy) were enrolled in the study.

The exclusion criteria for this study were pregnancy, hepatocellular carcinoma, portal vein thrombosis, grade III-IV hepatic encephalopathy, a history of treatment with calcium channel blockers, statin use during the previous 3 months, and a known allergy to any statin.

The patients in the study were randomized to either receive or not receive simvastatin. group I included 20 patients with cirrhosis who were administered 20 mg of simvastatin daily for 2 weeks followed by 40 mg of simvastatin daily for another 2 weeks, and group II included 20 patients who did not receive simvastatin as a control group. The included patients received their routine treatments of diuretics, liver support, and anti-diabetic or anti-hypertensive therapies during the study.

All patients provided detailed history including age, sex, residence, job, marital status, special habits, history of diabetes mellitus or anti-diabetic therapy, hypertension, anti-hypertension therapy, history of surgical shunts, history of gastrointestinal bleeding, history of upper endoscopy, and bleeding varices. A thorough clinical examination was conducted to assess for liver or spleen ascites, lower limb edema, jaundice, and hepatic encephalopathy.

All patients underwent routine laboratory investigations in our Tropical Medicine Clinic, including complete blood count (hemoglobin level, platelet count, and white blood count), kidney function tests (blood urea and serum creatinine), liver function profile (liver enzyme and total serum bilirubin levels, which were also measured 2 and 4 weeks after the beginning of the study to exclude any increase from baseline in group I), coagulation profile (international normalization ratio [INR], prothrombin time [PT], and prothrombin activity), random blood sugar (this was also measured 2 and 4 weeks after the beginning of the study to exclude any increase from baseline in group I), Child–Pugh score (assessed in all studied patients), and serum alpha-fetoprotein level.

Abdominal ultrasound was performed on all patients in the study to assess the liver (echogenicity, surface, edge, and size), attenuation of hepatic veins, spleen size, and presence of ascites.

Doppler ultrasound was performed on all patients to measure the portal vein diameter (PVD), portal vein velocity (PVV), portal vein blood flow (PVBF), portal vein flow direction, hepatic artery resistance index and hepatic artery pulsatility index (HARI and HAPI, respectively), splenic artery resistance index (SARI), portal hypertension index (PHI), and modified liver vascular index and liver vascular index (MLVI and LVI, respectively).

Doppler analysis was performed during quiet respiration or while the patients held their breath ^15^. All parameters were measured twice, at the beginning and at the end of the study. We placed the Doppler gate in the hilum of the spleen and in the porta hepatis of the liver. The same observer usually unified the method for measuring each index to avoid interobserver variability and calculated the mean of 3 consecutive measurements.

PVD was measured from the hilar segment when crossing the inferior vena cava while the patient was in the recumbent supine position. It was recorded in millimeters.

PVBF was calculated automatically after recording the peak, lowest, and mean venous velocity of the flow and the measurement of a cross-sectional area of the vessel lumen in a transverse plane. It was recorded in liters per minute (L/min). Portal vein flow direction: the direction of portal blood flow was shown by color Doppler, indicating if it was toward (hepatopetal) or away from the liver (hepatofugal).

PVV was calculated automatically after measuring (Vmax) and (Vmin). It was recorded in centimeters per second (cm/sec).

HARI: The hepatic artery was evaluated by demonstrating the artery proper while crossing the portal vein. HARI was calculated automatically after measuring the hepatic artery peak velocity and end diastolic velocity measured in meters per second (m/sec) at the porta hepatis. The resistance index was calculated using the following equation: [peak systolic velocity (V max) - end diastolic velocity/peak systolic velocity (V min)/mean velocity] ^16^.

HAPI was calculated automatically using the following equation: [(V max) - (V min)/mean velocity] ^16^.

The resistance index and wave form of the right hepatic vein was measured as the maximum negative velocity - minimum negative velocity (or positive velocity in case of triphasic flow signal)/maximum negative velocity.

Hepatic vein waveforms were described as triphasic, biphasic, monophasic, or not assessed because of severe attenuation.

SARI: Color Doppler allowed identification of the main branches of the splenic artery by placing the transducer below the left costal margin ^17^. SARI was measured automatically after measuring (Vmax) and (Vmin), which were measured in meters per second (m/sec) by putting the cursor in the main branches of the splenic artery at the splenic hilum at the left intercostal space ^18^.

The resistance index was calculated using the following equation: [(Vmax) - (Vmin)]/peak systolic velocity] ^16^.

PHI was calculated as (HARI × 0.69) × (SARI × 0.87)/PVV ^19^. It was recorded in m/sec.

LVI was calculated as PVV/HAPI ^20, 21^. It was recorded in cm/sec.

MLVI was calculated as PVV/HARI ^20, 21^. It was recorded in cm/sec.

All abdominal ultrasound and Doppler ultrasound assessments were performed with a Toshiba Nemio XG apparatus (Toshiba, Japan) by using a 3.5 MHz convex probe with B-mode and color Doppler ultrasound in the Tropical Medicine Department. Before evaluation, the patients fasted for at least 6 hours. During the evaluation, the patients were in the supine position.

Dataset for Groups 1 and 2 of the study • N: Number
• Group: 1, 2
• Sex: •Male: 1 •Female: 2
• Previous portal hypertension-related GIT bleeding: •Yes: 1 •No: 0
• Endoscopy: •Yes: 1 •No: 0
• Varices and portal gastropathy: •Yes: 1 •No: 0
• History of β-blocker use: •Yes: 1 •No: 0
• Hypertension (HTN): •Yes: 1 •No: 0
• History of diuretic use: •Yes: 1 •No: 0
• Diabetes mellitus (DM): •Yes: 1 •No: 0
• Hepatic encephalopathy: •Yes: 1 •No: 0
• Hepatitis B virus (HBV): •Yes: 1 •No: 0
• Hepatitis C virus (HCV): • Yes: 1• NO: 0
• Jaundice: •Yes: 1 •No: 0
• Lower limb (LL) edema: •Yes: 1 •NO: 0
• Ascites: •Yes: 1 No: 0
• Child–Pugh class: •Child–Pugh class A: 1 •Child–Pugh class B: 2. •Child–Pugh class C: 3
• Myalgia: •Yes: 1 •No: 0
• Diarrhea: •Yes: 1 •No: 0
• Worsening of ascites: •Yes: 1 •No: 0
• Observed improvement in muscle cramps: •Yes: 1 •No: 0
• ALT Alanine transferase
• AST Aspartate aminotransferase
• HAPI Hepatic artery pulsatility index
• HARI Hepatic artery resistance index
• Hb Hemoglobin
• LVI Liver vascular index
• MLVI Modified liver vascular index
• PHI Portal hypertension index
• PVD Portal vein diameter
• PVBF Portal vein blood flow
• PVV Portal vein velocity
• SARI Splenic artery resistance index
• RBCs Red blood cells
• WBCs White blood cells Click here for additional data file. Copyright: © 2018 Elwan N et al. 2018 Data associated with the article are available under the terms of the Creative Commons Zero "No rights reserved" data waiver (CC0 1.0 Public domain dedication).

### Statistical Analysis

The Statistical Package for the Social Sciences software (version 19, IBM Corp., Armonk, NY) was used for statistical analysis after organization and tabulation of our data. The mean and standard deviation were used for numerical variables. Additionally, the t-test and paired t-test were used for comparison of mean values between groups. Differences in mean values between the four variables studied were tested using analysis of variance (ANOVA). When the value of ANOVA (F) was significant, Tukey’s test was used. Percentages, numbers, and the chi square test were used for categorical variables. The P value was considered non-significant if it was > 0.05, significant if it was < 0.05, and highly significant if it was < 0.001).

## Results

Forty patients with cirrhosis and PHT were enrolled from April to November 2016 (38 were hepatitis C positive, 1 was hepatitis B positive, and 1 had combined hepatitis B and C infection). There was no statistically significant difference between the two groups with regard to age, sex, clinical features, medical history (hypertension, diabetes mellitus, and history of diuretics therapy), or Child–Pugh classification ( Table 1, Table 2).

**Table 1.** Baseline demographic and clinical features of the studied groups.

	Group I (n = 20) No. %	Group II (n = 20) No. %	χ ^2^	P value
**Age**	51.5 ± 6.692	50.8 ± 6.993	0.323	0.748
**Sex:** **Male:** **Female:**	10 50.0 10 50.0	16 80.0 4 20.0	**χ** ^2^ = 2.747	0.097
**Jaundice:**	18 90.0	19 95.0	0.360	0.548
**Ascites:**	15 75.0	16 80.0	0.143	0.705
**Encephalopathy:**	7 35.0	4 20.0	1.129	0.288
**LL edema:**	17 85.0	18 90.0	0.229	0.633
**HTN:**	3 15.0	1 5.0	1.111	0.292
**DM:**	4 20.0	3 15.0	0.173	0.677
**History of upper endoscopy with** **varices**	16 80.0	14 70.0	0.533	0.465
**Previous portal hypertension related** **GI bleeding**	2 10.0	1 5.0	0.360	0.548
**Child–Pugh class:** **A:** **B:** **C:**	3 15.0 12 60.0 5 25.0	1 5.0 9 45.0 10 50.0	3.095	0.213

DM: Diabetes mellitus, GI: Gastrointestinal, HTN: Hypertension, LL: Lower leg Regarding laboratory investigations, there was no significant difference between the two groups.

**Table 2.** Baseline laboratory data of the studied groups.

	Group I (n = 20) No. %	Group II (n = 20) No. %	T	P value
**Hb** **(12-16 g/dL)**	10.715 ± 1.523	10.175 ± 1.011	1.321	0.194
**Platelets (150-** **450 × 10 ^3^ µL)**	104.40 ± 23.90	101.30 ± 29.07	0.362	0.719
**WBCs** **(4-11 × 10 ^3^/mm ^3^)**	4.785 ± 1.830	4.070 ± 1.484	1.357	0.183
**AST**	64.900 ± 29.704	51.750 ± 33.488	1.321	0.194
**ALT**	40.300 ± 17.336	39.400 ± 32.531	1.674	0.102
**Serum albumin** **(3.5-5.5 gm/dL)**	2.780 ± 0.509	2.710 ± 0.434	0.362	0.719
**Total serum** **bilirubin** **(0.2-1.2 mg/dL)**	4.785 ± 1.830	2.660 ± 1.371	1.357	0.183
**Serum creatinine** **(0.2-1.2 mg/dL)**	0.930 ± 0.283	1.040 ± 0.266	-1.266	0.213

ALT: Alanine transferase, AST: Aspartate aminotransferase, Hb: Hemoglobin, WBCs: White blood cells

Regarding the Doppler parameters of the studied groups ( Table 3), there was a significant difference in PVD between groups I and II at baseline (13.210 ± 2.353 mm vs. 14.805 ± 2.528 mm; P = 0.046). There was no difference between PVD at baseline and PVD after 30 days in both groups.

**Table 3.** Doppler parameters of the studied groups.

	Group I	Group II	T	P value
**PVD (Normal < 13 mm)**				
**Baseline**	**13.210 ± 2.353**	**14.805 ± 2.528**	**-2.065**	0.046*
**30 days after**	**13.440 ± 2.204**	**14.265 ± 2.209**	**-1.182**	**0.244**
**P value**	**0.306**	**0.192**		
**PVV (15.5 ± 4.0 cm/sec)**				
**Baseline**	**9.025 ± 2.400**	**8.915 ± 2.199**	0.151	0.881
**30 days after**	**9.470 ± 2.202**	**8.513 ± 2.714**	1.225	0.228
**P value**	**0.358**	**0.424**		
**PVFV (0.864 ± 0.188** **L/min)**				
**Baseline**	**0.548 ± 0.284**	**0.557 ± 0.358**	-0.088	0.930
**30 days after**	**0.641 ± 0.367**	**0.510 ± 0.338**	1.175	0.247
**P value**	**0.180**	**0.630**		
**HARI (0.55–0.7)**				
**Baseline**	**0.785 ± 0.088**	**0.739 ± 0.079**	1.748	0.088
**30 days after**	**0.717 ± 0.086**	**0.757 ± 0.088**	-1.455	0.154
**P value**	< 0.001*	**0.478**		
**HAPI (0.92 ± 0.1)**				
**Baseline**	**1.544 ± 0.553**	**1.524 ± 0.403**	0.134	0.894
**30 days after**	**1.410 ± 0.348**	**1.609 ± 0.446**	-1.573	0.124
**P value**	**0.064**	**0.379**		
**PHI (1.393 ± 0.52 m/sec)**				
**Baseline**	**3.915 ± 0.973**	**3.080 ± 0.610**	2.827	0.007*
**30 days after**	**3.605 ± 1.168**	**3.000 ± 0.858**	1.867	0.070
**P value**	0.024*	**0.528**		
**LVI (11.71 ± 2.9 cm/sec)**				
**Baseline**	**6.420 ± 2.561**	**6.140 ± 2.011**	0.385	0.703
**30 days after**	**7.094 ± 2.135**	**5.630 ± 2.640**	1.928	0.061
**P value**	**0.188**	**0.280**		
**MLVI (43.8 ± 7.2 cm/sec)**				
**Baseline**	**11.540 ± 3.266**	**12.170 ± 3.506**	-0.588	0.560
**30 days after**	**13.305 ± 3.222**	**11.000 ± 2.968**	2.353	0.024*
**P value**	0.009*	**0.100**		
**SARI (0.57 ± 0.04)**				
**Baseline**	**0.697 ± 0.073**	**0.609 ± 0.101**	3.177	0.003*
**30 days after**	**0.668 ± 0.083**	**0.615 ± 0.096**	1.853	0.072
**P value**	**0.231**	**0.767**		

PVD: Portal vein diameter, PVV: Portal vein velocity, PVFV, HARI: Hepatic artery resistance index, HAPI: Hepatic artery pulsatility index, PHI: Portal hypertension index, LVI: Liver vascular index, MLVI: Modified liver vascular index, SARI: Splenic artery resistance index

There was no significant difference in the baseline mean values of PVV, PVBF, and HAPI between groups I and II (P = 0.881, 0.930, and 0.894, respectively). No difference was detected between baseline measurements and the measurements taken after simvastatin administration for 30 days in group I (P values: 0.358, 0.180, and 0.064, respectively).

There was no significant difference in HARI at baseline between groups I and II (0.785 ± 0.088 vs. 0.739 ± 0.079; P = 0.088). There was a highly significant decrease in HARI after simvastatin administration for 30 days in group I, from 0.785 ± 0.088 to 0.717 ± 0.086 (P < 0.001), an estimated 8.7% decrease from baseline.

PHI was significantly higher in group I than in group II at baseline (3.915 ± 0.973 m/sec vs. 3.080 ± 0.610 m/sec; P = 0.007). PHI was significantly decreased in group I after simvastatin administration for 30 days, from 3.915 ± 0.973 m/se to 3.605 ± 1.168 m/sec (P = 0.024), a 7.9% decrease from baseline.

The mean LVI at baseline was 6.420 ± 2.561 cm/sec in group I and 6.140 ± 2.011 cm/sec in group II with no significant difference (P = 0.703). LVI was 6.420 ± 2.561 cm/sec at baseline and 7.094 ± 2.135 cm/sec after 30 days of simvastatin administration with no difference (P = 0.188).

The mean MLVI at baseline was 11.540 ± 3.266 cm/sec and 12.170 ± 3.506 cm/sec in groups I and II, respectively, with no significant difference (P = 0.560). MLVI was significantly increased in group I after simvastatin administration for 30 days, from 11.540 ± 3.266 cm/sec to 13.305 ± 3.222 cm/sec (P = 0.009), an increase of 15.3% from baseline. The MLVI was significantly higher in group I, after simvastatin administration for 30 days, than in group II (13.305 ± 3.222 cm/sec vs. 11.000 ± 2.968 cm/sec; P = 0.024).

The mean SARI value was 0.697 ± 0.073 and 0.609 ± 0.101 in groups I and II, respectively, with a significant difference between them (P = 0.003). There was no significant difference between SARI at baseline and after simvastatin administration for 30 days in group I (0.697 ± 0.073 vs. 0.668 ± 0.083; P = 0.23).

**Figure 1.**
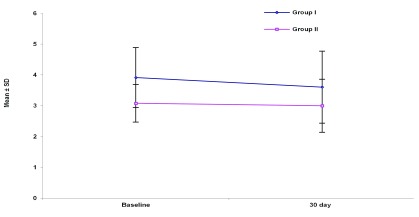
Portal hypertension index (PHI) in the studied groups.

PHI decreased significantly in group I after simvastatin administration for 30 days, from 3.915 ± 0.973 m/sec to 3.605 ± 1.168 m/sec (P = 0.024), a decrease of 7.9% from baseline ( Figure 1).

No significant difference was found in the adverse effects noted during the study between the 2 groups in terms of myalgia, muscle pain, diarrhea, or worsening of ascites. The incidence of myalgia or muscle pain was reported to be 10% in group I and 20% group II (P = 0.376). The incidence of diarrhea was reported to be 10% in group I and 5% in group II (P = 0.548). The incidence of worsening ascites was reported to be 15% in group I and 25% in group II (P = 0.429) ( Table 4).

**Table 4.** Adverse effects detected during the course of the study.

Adverse effects		Groups						Chi-Square	
		Group I (N = 20)		Group II (N = 20)		Total			
		N	%	N	%	N	%	χ ^2^	P-value
**Myalgia or** **muscle pain**	**No**	18	90.00	16	80.00	34	85.00	0.784	0.376
	**Yes**	2	10.00	4	20.00	6	15.00		
**Diarrhea**	**No**	18	90.00	19	95.00	37	92.50	0.360	0.548
	**Yes**	2	10.00	1	5.00	3	7.50		
**Worsening** **of ascites**	**No**	17	85.00	15	75.00	32	80.00	0.625	0.429
	**Yes**	3	15.00	5	25.00	8	20.00		

## Discussion

PHT is considered to be an inevitable outcome of liver cirrhosis ^1^. We conducted a randomized controlled clinical trial to study PHT by Doppler ultrasound in patients with cirrhosis prior to and after receiving simvastatin, and we studied other Doppler parameters reflecting hepatic fibrosis in patients with cirrhosis.

There was no significant difference regarding age, sex, or Child–Pugh score between the two studied groups, but there was a significant difference between the two groups with regard to INR, PT, and prothrombin activity at baseline. INR and PT were significantly higher, while prothrombin activity was significantly lower in group II than in group I. This may be because 50% of the patients in group II had Child–Pugh class C with advanced cirrhosis, and 45% had Child–Pugh class B cirrhosis. This was in accordance with the findings of Schuppan and Afdhal ^22^, who reported that PT is increased in patients with advanced cirrhosis, as those patients have significantly impaired synthetic function.

Before simvastatin treatment, PVD was significantly higher in group II than in group I, which might be related to the severity of liver disease. This was in accordance with the findings of Shateria *et al.* ^23^. In contrast, Ong and Tan ^24^ reported that PVD does not correlate with high portal pressure or cirrhosis severity, while Lafortune *et al.* ^25^ concluded that PVD might even decrease with an increase in PHT severity.

We also found that the mean PVV and mean PVBF were lower than normal in both groups. This was similar to the findings of Al-Nakshabandi ^26^, who reported that a flow velocity of <16 cm/sec is a diagnostic feature of PHT in patients with cirrhosis. This decrease in PVV may be due to the presence of PHT, which results in increasing resistance to portal blood flow ^27^. This was in accordance with the findings of Achim *et al.* ^28^. They compared the mean PVBF and PVV between patients with cirrhosis and a healthy control group and found that these parameters were significantly lower in patients with cirrhosis, and this decrease was more notable in patients with Child–Pugh class B and C.

Furthermore, HARI and HAPI were higher than normal. This may be due to the increase in hepatic arterial vascular resistance parallel to the rise in the portal pressure ^29^. Additionally, it may be explained by the hepatic artery buffer response mechanism ^30^.

The results of this study were similar to those of other studies ^28, 31^, which reported that HARI was higher in patients with cirrhosis and PHT than in control subjects, and the findings of Zhang *et al.* ^32^, who concluded that HAPI was higher in patients than in controls and that portal pressure was significantly positively correlated with HAPI.

## Conclusions

In conclusion, simvastatin significantly decreased PHI and HARI in patients with cirrhosis and PHT. Moreover, simvastatin significantly improved liver perfusion, as shown by the increased MLVI in patients with cirrhosis and PHT. These effects were achieved with or without the administration of β-adrenergic blockers. Therefore, simvastatin could be a valuable therapy for PHT, as simvastatin administration was associated with lowered hepatic resistance without harmful effects on systemic circulation. Additionally, simvastatin is relatively safe for patients with cirrhosis and PHT whether compensated or not.

## Data Availability

The data referenced by this article are under copyright with the following copyright statement: Copyright: © 2018 Elwan N et al.

Data associated with the article are available under the terms of the Creative Commons Zero "No rights reserved" data waiver (CC0 1.0 Public domain dedication). 

Dataset 1: Dataset for Groups 1 and 2 of the study 10.5256/f1000research.13915.d195997 ^33^

• N: Number
• Group: 1, 2
• Sex: •Male: 1 •Female: 2
• Previous portal hypertension-related GIT bleeding: •Yes: 1 •No: 0
• Endoscopy: •Yes: 1 •No: 0
• Varices and portal gastropathy: •Yes: 1 •No: 0
• History of β-blocker use: •Yes: 1 •No: 0
• Hypertension (HTN): •Yes: 1 •No: 0
• History of diuretic use: •Yes: 1 •No: 0
• Diabetes mellitus (DM): •Yes: 1 •No: 0
• Hepatic encephalopathy: •Yes: 1 •No: 0
• Hepatitis B virus (HBV): •Yes: 1 •No: 0
• Hepatitis C virus (HCV): • Yes: 1• NO: 0
• Jaundice: •Yes: 1 •No: 0
• Lower limb (LL) edema: •Yes: 1 •NO: 0
• Ascites: •Yes: 1 No: 0
• Child–Pugh class: •Child–Pugh class A: 1 •Child–Pugh class B: 2. •Child–Pugh class C: 3
• Myalgia: •Yes: 1 •No: 0
• Diarrhea: •Yes: 1 •No: 0
• Worsening of ascites: •Yes: 1 •No: 0
• Observed improvement in muscle cramps: •Yes: 1 •No: 0
• ALT Alanine transferase
• AST Aspartate aminotransferase
• HAPI Hepatic artery pulsatility index
• HARI Hepatic artery resistance index
• Hb Hemoglobin
• LVI Liver vascular index
• MLVI Modified liver vascular index
• PHI Portal hypertension index
• PVD Portal vein diameter
• PVBF Portal vein blood flow
• PVV Portal vein velocity
• SARI Splenic artery resistance index
• RBCs Red blood cells
• WBCs White blood cells

## Ethics and consent

All patients provided written informed consent, and the study was approved by the Ethics Committee of the Faculty of Medicine, Tanta University. All patients had code numbers to ensure patient anonymity.

## List of abbreviations

**Table:** 

*ALT*	Alanine transferase
*AST*	Aspartate aminotransferase
*HAPI*	Hepatic artery pulsatility index
*HARI*	Hepatic artery resistance index
*Hb*	Hemoglobin
	s
*INR*	International normalization ratio
*LVI*	Liver vascular index
*MLVI*	Modified liver vascular index
*n*	Number
*PHI*	Portal hypertension index
*PHT*	Portal hypertension
*PT*	Prothrombin time
*PVD*	Portal vein diameter
*PVBF*	Portal vein blood flow
*PVV*	Portal vein velocity
*SARI*	Splenic artery resistance index
	g
*WBCs*	White blood cells

## Supplementary Material

Supplementary file 1: CONSORT flow diagram

Supplementary file 1: CONSORT check list

Supplementary file 1: Study protocol
