# Supplementary material for: Evaluation of portal pressure by doppler ultrasound in patients with cirrhosis before and after simvastatin administration – a randomized controlled trial
Source: F1000Res. 2018 Mar 1;7:256. [Version 1] doi: 10.12688/f1000research.13915.1 (PMC5883384; doi:10.12688/f1000research.13915.1)
Supplement: Supplementary file 2 [file f1000research-7-15128-s0001.tgz › a5bf5c88-4475-49a0-a450-0e887afc879a.docx]

Assessed for eligibility (n = 47)

Excluded (n = 7)

Did not meet inclusion criteria (n = 5)

Other causes (n = 2)

Randomized (n = 40)

Allocation

20 patients who did not receive simvastatin

20 patients received simvastatin 20 mg/day for 2 weeks, increased to 40 mg/day on day 15 for another 2 weeks

Analysis

Analyzed (n = 20)

Analyzed (n = 20)

No patient was lost to follow up

All patients completed the course of treatment

Follow up
