# Supplementary material for: Evaluation of portal pressure by doppler ultrasound in patients with cirrhosis before and after simvastatin administration – a randomized controlled trial
Source: F1000Res. 2018 Mar 1;7:256. [Version 1] doi: 10.12688/f1000research.13915.1 (PMC5883384; doi:10.12688/f1000research.13915.1)
Supplement: Supplementary file 4 [file f1000research-7-15128-s0003.tgz › 87d60e19-54bc-459c-9fac-e8f2b39d8223.docx]

**Evaluation of Portal Pressure by Doppler Ultrasound in Patients with Cirrhosis Before and After Simvastatin Administration – A Randomized Controlled Trial**

Sponsor: Tanta University

Information provided by (Responsible Party): Tanta University

Study Description

Brief Summary:

Portal hypertension itself is not a disease. Rather, it is an indication of a condition caused mostly by chronic lesions of the liver with distinct causes, such as viral infection, chronic alcoholism, or metabolic disorders. Other reasons include splanchnic vascular diseases (for example, obstruction of the portal or hepatic veins). Portal hypertension is defined as pressure in the portal vein exceeding the vena cava pressure by more than 5 mm Hg.

| **Condition or disease** | **Intervention/treatment** | **Phase** |
| --- | --- | --- |
| Portal hypertension | Drug: simvastatin, Other: not received simvastatin | Phase 4 |

Detailed Description:

The use of simvastatin might attenuate liver fibrosis in patients with chronic hepatitis C infection; it may also reduce hepatic vascular resistance and portal pressure by improving liver generation of nitric oxide and hepatic endothelial dysfunction in patients with cirrhosis; therefore, it could be an effective therapy for portal hypertension. It might even improve the survival of patients with cirrhosis after variceal bleeding.

Study Design

|  |  |
| --- | --- |
| Study Type: | Interventional (Clinical Trial) |
| Estimated Enrollment: | 40 participants |
| Allocation: | Randomized |
| Intervention Model: | Parallel Assignment |
| Masking: | None (Open Label) |
| Primary Purpose: | Treatment |
| Official Title: | Evaluation of Portal Pressure by Doppler Ultrasound in Patients with Cirrhosis Before and After Simvastatin Administration – A Randomized Controlled Trial |
| Study Start Date: | October 2016 |
| Estimated Primary Completion Date: | December 2018 |
| Estimated Study Completion Date: | December 2018 |

**Resource links provided by the National Library of Medicine**

[Drug Information](https://druginfo.nlm.nih.gov/drugportal) available for: [simvastatin](https://druginfo.nlm.nih.gov/drugportal/name/Simvastatin)

[U.S. FDA Resources](https://clinicaltrials.gov/ct2/info/fdalinks)

Arms and Interventions

| **Arm** | **Intervention/treatment** |
| --- | --- |
| Active Comparator: Simvastatin  Simvastatin 20 mg/day for 2 weeks, increased to 40 mg/day on day 15 for another 2 weeks | Drug: Simvastatin  Simvastatin 20 mg/day for 2 weeks increased to 40 mg/day on day 15 for another 2 weeks plus the routine treatment  Other Name: Corvast |
| Placebo Comparator: Placebo  Placebo | Other: Placebo  Placebo |

Outcome Measures

Primary Outcome Measures:

1. Number of patients with reduced portal pressure after intervention [Time frame: 6 months]

The number of patients with reduced portal pressure after intervention

Eligibility Criteria

|  |
| --- |

Inclusion Criteria:

- Positive diagnosis of cirrhosis by ultrasound (coarse echogenic pattern, bulky caudate lobe, attenuated hepatic veins)
- Clinical manifestations of portal hypertension (as esophageal varices, splenomegaly, ascites, and grade I-II encephalopathy)

Exclusion Criteria:

- Pregnancy
- Grade III-IV hepatic encephalopathy
- Hepatocellular carcinoma
- Portal vein thrombosis.
- Statin use in the previous 3 months
- Hypersensitivity to statins
- Previous surgical shunt or tansjugular portosystemic shunt TIPS
- History of treatment with calcium channel blockers

Contacts and Locations

**Information from the National Library of Medicine**
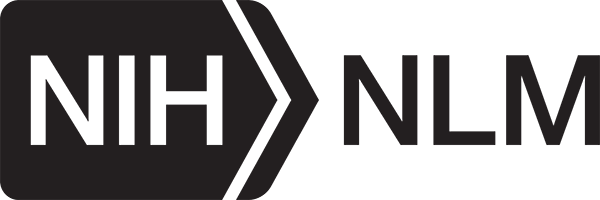


*To learn more about this study, you or your doctor may contact the study research staff using the contact information provided by the sponsor.*

*Please refer to this study by its ClinicalTrials.gov identifier (NCT number):* ***NCT02994485***

Contacts

|  |  |  |  |
| --- | --- | --- | --- |
| Contact: Sherief Abd-Elsalam, CONSULTANT | 00201095159522 | [sheriefabdelsalam@yahoo.com](mailto:sheriefabdelsalam%40yahoo.com?subject=NCT02994485,%20Nadia%20Elwan,%20Evaluation%20Of%20The%20Portal%20Pressure%20By%20Doppler%20Ultrasound%20In%20Cirrhotic%20Patients%20Before%20And%20After%20Simvastatin) |  |

Locations

|  |  |
| --- | --- |
| Egypt | |
| Sherief Abd-Elsalam | Recruiting |
| Tanta, Egypt | |
| Contact: Sherief Abd-Elsalam, Lecturer    00201000040794    [Sherif_tropical@yahoo.com](mailto:Sherif_tropical%40yahoo.com?subject=NCT02994485,%20Nadia%20Elwan,%20Evaluation%20Of%20The%20Portal%20Pressure%20By%20Doppler%20Ultrasound%20In%20Cirrhotic%20Patients%20Before%20And%20After%20Simvastatin) | |
