# Supplementary material for: Evaluation of portal pressure by doppler ultrasound in patients with cirrhosis before and after simvastatin administration – a randomized controlled trial
Source: F1000Res. 2018 Mar 1;7:256. [Version 1] doi: 10.12688/f1000research.13915.1 (PMC5883384; doi:10.12688/f1000research.13915.1)
Supplement: Peer review history

# Peer review: Referee response for version 1

**DOI:** 10.5256/f1000research.15128.r31479

**Published:** 2018-04-03

**Authors:** Abidullah Khan

**Affiliations:** 1 Department of Medicine, Khyber Teaching Hospital, Peshawar, Pakistan

I congratulate Elwan N et al for their efforts. The research ideas being brought forward in this manuscript are worth appreciation. However, the authors need to address the following deficiencies before any consideration for final publication is made.

 1) TITLE:

 This needs to be modified. I will suggest to rephrase it as, “ Effects of Simvastatin on portal pressure in patients with cirrhosis”.

 2) ABSTRACT:

 Please avoid using abbreviations in the abstract. Moreover, abbreviations need to be written in full on their first appearance in a given article. In my opinion, the term PHT should therefore be expanded.

 The term “hypotensive agents” must be rephrased as “one of the portal hypotensive agents”.

 3) INTRODUCTION:

 In para 4, the authors write that simvastatin can be used safely in patients with chronic liver disease. I will need an explanation for this as at what stage can it be more effective and safer?  This is because, advanced liver disease in itself leads to hypolipidemia which in itself is a worse prognostic indicator.

 4) METHODS:

 The sample size is small. I will recommend more participants.

 Regarding group 1, why were they segregated into two subgroups in terms of the dosage of simvastatin? If they were done so, why were the effects of the dosage on portal pressure not provided in the results/discussion sections? Moreover, was there any difference in the final outcomes because of the different dosages?

 The authors write that the patients were continued on their usual antihypertensive and diabetic medications. Were any potential drug-drug interactions excluded? Moreover, I will be interested  in knowing the antihypertensive medications (esp ACEi/ARBs as they have antifibrotic and anti inflammatory effects).

 5) RESULTS:

 Th authors mention that all the patients included in the study had cirrhosis secondary to chronic viral hepatitis. Hence, the results can not be generalized to all forms of cirrhosis and either the title needs to be modified accordingly or more patients need to be recruited to better measure the etiology-outcome effect.

 In para 2, PVD in group 1 was 13.2 in contrast  to group 2 where it was 14.8. Hence, there was a difference at baseline. This contradicts their statement that there was no difference in mean PVDs at baseline.

 6) DISCUSSION:

 The authors are requested to avoid repetition of the facts already mentioned in Introduction and Methods etc. The para 1 of discussion can therefore be either modified or removed.

 In para 3, the authors mention that PVD was higher in group 2 than in group 1. This is against their statement in Results as explained  above. Moreover, as group 1 had a lower PVD at baseline, can the lowering effect of simvastatin be purely a chance finding? Therefore, it is suggested to include more patients in the study group.

 7) CONCLUSION:

 Please make the conclusion more succinct and comprehensive.

 Thank you.

I have read this submission. I believe that I have an appropriate level of expertise to confirm that it is of an acceptable scientific standard, however I have significant reservations, as outlined above.

# Peer review: Referee response for version 1

**DOI:** 10.5256/f1000research.15128.r31373

**Published:** 2018-03-16

**Authors:** Mohamed Ahmed Samy Kohla

**Affiliations:** 1 Department of Hepatology, National Liver Institute, Menoufia University, Shebin Al-Kom, Egypt

The study entitled: Evaluation of portal pressure by doppler ultrasound in patients with cirrhosis before and after simvastatin administration – a randomized controlled trial, is quite interesting and of clinical significance which could have a good impact on treatment of portal hypertension with Simvastatin, which  is a safe and well tolerated drug.

 However, I have few comments:

1. I think this study actually aims at evaluation of portal hemodynamics rather than portal pressure, because Doppler U/S can asses portal hemodynamics rather than measurement of portal pressure,therefore, i suggest a minor modification of the title:
Evaluation of portal hemodynamics by doppler ultrasound in patients with cirrhosis before and after simvastatin administration – a randomized controlled trial
2. Data on MELD score should be provided, we can compare the mean MELD score in the 2 groups.
3. Did all patients in the 2 groups had an upper GI endoscopy before enrollment? If yes, the endoscopic findings should be shown to asses varices and portal hypertensive gastropathy.
4. In the method section, the authors mentioned measurement of Random blood sugar and AFP but this was not shown in the tables.
5. Were patients on beta blockers excluded from the study? Was there a drug free interval before enrollment?
6. Why was the dose of Simvastatin escalated from 20 mg to 40 mg per day after 2 weeks?

My recommendation is that, this manuscript is suitable for publication (accepted with minor changes).

I have read this submission. I believe that I have an appropriate level of expertise to confirm that it is of an acceptable scientific standard.

# Peer review: Referee response for version 1

**DOI:** 10.5256/f1000research.15128.r31377

**Published:** 2018-03-07

**Authors:** William Beaubien-Souligny

**Affiliations:** 1 Department of Anesthesiology and Intensive Care, Montreal Heart Institute, Montréal, QC, Canada; 2 Department of Nephrology, Centre Hospitalier Universitaire de Montréal, Montréal, QC, Canada

I had the opportunity to review the manuscript entitled “Evaluation of portal pressure by Doppler ultrasound in patients with cirrhosis before and after simvastatin administration – a randomized controlled trial.” In this project, the authors performed ultrasound assessment to determine whether treatment with simvastatin resulted in findings suggestive of a decrease in portal vein pressure compared to placebo.

 The subject is of interest for clinicians involved in the care of cirrhosis patients, particularly as the studied agent is already widely available. While these data are clinically relevant and must be reported, I have numerous concerns about data reporting in this work. I will list the main issues that must be addressed in my opinion, followed by other minor points.

 Main concerns:

1. As a randomized controlled trial, the comparator is the placebo group (as it was written in the Clinicaltrials.gov entry). It is clear, just by reading the abstract of this article, that the results are not adequately reported. Group 1 and group 2 (placebo) are not compared in the result section of the abstract: the main result reported is a pre-post analysis without comparison with the control group. The main analysis of your study should be as described in your clinicaltrials.gov entry: comparing the intervention and the placebo arm on the primary outcome (The number of patients with reduced portal pressure after intervention). It is also important clearly determine what was the primary outcome of the study in the method section (what ultrasound parameter is the most clinically important for the evaluation of portal hypertension). Other less important echographic parameters should be considered secondary outcomes.
2. In order for the readers to adequately assess the risk of bias in this study, I think it is important for the authors to give information about the following points which are included in the Cochrane Risk of Bias assessment tool:
  - Please describe the method of randomization
  - Please describe the method of allocation concealment (if any)
  - Please describe if there was blinding during outcome assessment (does the ultrasonographer knew patient allocation to one arm or the other)
  - There was no study protocol available for review. The file identified as the study protocol was a copy of the NCT entry and thus was insufficient. I think points a and b are especially important as baseline differences were seen between the intervention and the placebo arm (more severe cirrhosis in the intervention arm)
3. The discussion section should be heavily modified. The authors do not discuss previous clinical trials of the subject. There is at least 4 previous trials on the subject. (PMID: 29099421, 26321186, 26774179, 19208350) This is a vital part of the discussion section that must highlight how this work compares with previous studies. Additionally, the limitations of the study are not discussed which is usually an integral part of the discussion section. 

Other specific comments:

- Abstract: Please see main concern no 1
- Methods
  - Please clarify the inclusion criteria for portal hypertension. Were all the features required to be present or only one of them was sufficient?
  - How were patients approached for this study? On an outpatient basis or during hospitalisation?
  - Please make sure to indicate that this is a open-label study both in the abstract and in the method.
  - Please describe how you determined that the distribution for the continuous variables was normal
  - Please describe how you determined sample size
- Results:
  - Too much emphasis is put on the baseline values. I don’t think it is necessary to repeat what is found in the Tables except for clinically or statistically significant differences between groups.
  - Focus on the comparison of outcome between the intervention and the control group. Pre-post analysis should be secondary in a randomized clinical trial.
  - How many patients were on nadolol. Were the dose modified during the treatment period?
- Discussion
  - Please see main concern no 3
  - Please provide a small summary of the main findings at the beginning.
  - The discussion about echographic parameters is of limited interest in its present form. I would suggest to focus of the clinical implications of these findings. Surely, you think that some of those markers represent good surrogates for the severity of portal hypertension and may be used as a pharmacologic treatment target? This should represent an opportunity to describe what is the best marker (primary outcome) and if it was modified by statin therapy.
  - It should be discussed in the results that the baseline imbalance in PHI between the intervention and control group is problematic in this study and probably affect the lack of difference between the two groups at 30 days. Was this imbalance due to chance? Giving more information (see major point 2) would help the reader to understand the possible explanations.

 

 I will be available to reconsider this manuscript after these issues are addressed. Thank you for the opportunity to review this work.

 Sincerely

 William Beaubien-Souligny MD FRCPC

I have read this submission. I believe that I have an appropriate level of expertise to confirm that it is of an acceptable scientific standard, however I have significant reservations, as outlined above.

## References

[1] BishnuSAhammedSMSarkarAHembramJChatterjeeSDasKDhaliGKChowdhuryADasK: Effects of atorvastatin on portal hemodynamics and clinical outcomes in patients with cirrhosis with portal hypertension: a proof-of-concept study. *Eur J Gastroenterol Hepatol*.2018;30(1) : 10.1097/MEG.0000000000001006 54-59 10.1097/MEG.0000000000001006 29099421

[2] Pollo-FloresPSoldanMSantosUCKunzDGMattosDEda SilvaACMarchioriRCRezendeGF: Three months of simvastatin therapy vs. placebo for severe portal hypertension in cirrhosis: A randomized controlled trial. *Dig Liver Dis*.2015;47(11) : 10.1016/j.dld.2015.07.156 957-63 10.1016/j.dld.2015.07.156 26321186

[3] AbraldesJGVillanuevaCAracilCTurnesJHernandez-GuerraMGenescaJRodriguezMCastelloteJGarcía-PagánJCTorresFCallejaJLAlbillosABoschJBLEPS Study Group: Addition of Simvastatin to Standard Therapy for the Prevention of Variceal Rebleeding Does Not Reduce Rebleeding but Increases Survival in Patients With Cirrhosis. *Gastroenterology*.150(5) : 10.1053/j.gastro.2016.01.004 1160-1170.e3 10.1053/j.gastro.2016.01.004 26774179

[4] AbraldesJGAlbillosABañaresRTurnesJGonzálezRGarcía-PagánJCBoschJ: Simvastatin lowers portal pressure in patients with cirrhosis and portal hypertension: a randomized controlled trial. *Gastroenterology*.2009;136(5) : 10.1053/j.gastro.2009.01.043 1651-8 10.1053/j.gastro.2009.01.043 19208350
